# The Xi'an Schizophrenia Imaging Lab (SIL) data and ten years of MRI study on schizophrenia

**DOI:** 10.1093/psyrad/kkac008

**Published:** 2022-08-11

**Authors:** Long-Biao Cui, Hong Yin

**Affiliations:** Schizophrenia Imaging Laboratory, and the Department of Clinical Psychology, Fourth Military Medical University, Xi'an 710032, China; Department of Radiology, Xi'an People's Hospital (Xi'an Fourth Hospital), Xi'an 710004, China

The Schizophrenia Imaging Laboratory (SIL) data are based on a collaboration of >10 years studying the schizophrenic brain using magnetic resonance imaging (MRI) in Xi'an, China. Collection of SIL data (*N* = 665; 319 patients, 48 first-degree relatives, and 298 control participants) started in 2011, with the purpose of performing a trans-scale study focusing on schizophrenia, and this has since diversified into three datasets: pooling clinical assessment, neuroimaging and genetic data to answer clinical and preclinical questions in psychiatry. Most of them come from Fourth Military Medical University, and the rest of the data come from the Xi'an Mental Health Center. In the SIL data, all the participants underwent clinical assessments (clinical characteristics, e.g. Positive and Negative Syndrome Scale, and cognitive tests) and MRI scans, including T2-weighted imaging, high-resolution T1-weighted imaging, functional imaging, diffusion weighted imaging, and arterial spin labeling at baseline, and 103 participants had transcriptome-wide data of whole blood (mRNA, small RNA, lncRNA, and circRNA). Scanning machines included the GE Discovery MR750 3.0 T scanner and Siemens 3.0 T Magnetom Trio Tim MR scanner. Clinical assessment at discharge from the hospital was available for 188 patients whose episode resulted in hospitalization. Afterward, 148 participants completed the follow-up assessments and scans. The whole study had no influence on the therapy. It investigated different aspects of familial risk, neural mechanisms, symptoms, diagnosis, treatment, and clinical translation.

Central to the success of these 10 years are the efforts of the dedicated staff at SIL. Importantly, this work was supported by the National Key Basic Research and Development Program (2011CB707805), National Natural Science Foundation (81571651, 81801675), project funding by the China Postdoctoral Science Foundation (2019TQ0130, 2020M683739), Fourth Military Medical University (2019CYJH, 2014D07), and the State Scholarship Fund, China Scholarship Council (201603170143).

The data set has been a formidable force for innovation in neuroimaging of schizophrenia. SIL data support >50 active studies. Summaries of these key studies are listed in Table 1. SIL consistently contributes to the characterization of robust neuroimaging phenotype, objective diagnosis, and therapeutic effects on brain and prediction of treatment outcomes. First, targeting the biological phenotype, we discovered new evidence of neurodevelopmental disorders associated with a disrupted brain connectome throughout the course, and established a new phenotype of auditory verbal hallucinations. Second, we explored new strategies in biological psychiatry for objective diagnosis, and creatively applied radiomics to identify this disease without solid lesions. Furthermore, to optimize treatment levels, we revealed the mechanism for predicting brain age by improved brain structural networks after antipsychotic treatment, and constructed a new model for efficacy prediction. SIL has worked with researchers to publish evidence that advances psychiatry, neuroscience, and radiology, and improves schizophrenia patient care. Requests to access SIL data and further inquiries can be directed to us.

**Table 1:** A selection of key findings using SIL data.

Publications	Total N from SIL data	Main findings
Xi *et al*., 2022	100 controls and 100 patients	Early medication improves the brain aging of patients with schizophrenia.
Li *et al*., 2020	54 controls and 90 patients	A neuroimaging biomarker based on functional striatal abnormalities is developed for schizophrenia identification, prognosis, and subtyping.
Cui *et al*., 2019	114 controls and 81 patients	Disrupted rich club organization and functional dynamics might be an early feature in the pathophysiology of schizophrenia.
Rozycki *et al*., 2018	24 controls and 18 patients	Structural MRI provides a robust and reproducible imaging signature of schizophrenia.
Cui *et al*., 2017	19 controls and 32 patients	Dysfunction in brain regions in schizophrenia patients with auditory verbal hallucinations are involved in auditory processing, language production and monitoring, and sensory information filtering.

## Conflict of Interest

The authors declare no conflict of interests.
